# Non-Intrusive Continuous Monitoring of Leaks for an In-Service Penstock

**DOI:** 10.3390/s24165182

**Published:** 2024-08-11

**Authors:** Marius Nati, Cristina Despina-Stoian, Dragos Nastasiu, Denis Stanescu, Angela Digulescu, Cornel Ioana, Vincent Nanchen

**Affiliations:** 1 Telecommunications Technology Department, Military Technical Academy “Ferdinand I”, 050141 Bucharest, Romania; alin.nati@mta.ro (M.N.); cristina.despina@mta.ro (C.D.-S.); dragos.nastasiu@mta.ro (D.N.); denis.stanescu@mta.ro (D.S.); angela.digulescu@mta.ro (A.D.); 2 Altrans Energies,38031 Grenoble, France; 3 GIPSA-Lab, UMR 5216 CNRS, Université Grenoble-Alpes, 38400 Grenoble, France; 4 OIKEN, 1951 Sion, Switzerland; vincent.nanchen@oiken.ch

**Keywords:** pipe leaks, non-intrusive monitoring, acoustic sensors, matched filter, phase diagram-based entropy

## Abstract

In modern industries, pipelines play a crucial role, both as an essential element in energy transportation (water, gas and electricity) and also in the distribution of these resources. The large size of piping infrastructures, their age and unpredictable external factors are the main difficulties in monitoring the piping system. In this context, the detection and the localization of leaks are challenging but essential, as leaks lead to substantial economic losses. Current methods have many limitations, involving invasive procedures, working only with short pipes or requiring a system shutdown. This paper presents a non-intrusive method based on acoustic signal processing. Leak detection is performed using matched filters, while localization is performed based on the phase diagram representation method and diagram-based entropy computation. Our continuous monitoring system was used for two months and a full comparison with the video inspection-based technique was conducted. The results indicate that this method has a high accuracy, regardless of the length of the pipe.

## 1. Introduction

Leaks from gas, water and oil pipelines cause serious problems, both economically and in terms of the health and safety of the population [1,2,3]. For these reasons, the need for effective methods for the detection and localization of leaks is an important current problem both from an industry and a research perspective [4,5,6,7]. Current methods involve either shutting down the system and emptying the pipelines in order to allow for video inspection, which requires a long period of downtime, or monitoring the pipelines using various imaging techniques, generally at high costs [8,9,10,11].

Transient Test-Based Techniques (TTBTs) present a method for detecting leaks in pipelines by exploiting the pressure waves that interact with potential leaks [12,13]. However, these techniques suffer from limitations such as the lack of continuous monitoring, invasiveness requiring direct intervention in pipeline systems, limited coverage, resource intensiveness and the risk of false positives/negatives [14,15]. To address these challenges, future directions should prioritize integrating TTBTs with continuous monitoring technologies, advancing non-invasive techniques, enhancing data analytics and interpretation and establishing standardized practices within the industry.

Another method of leak detection is based on acoustic transceivers. When gas/liquid is injected into the pipeline, the holes responsible for leaks produce an acoustic emission that is transmitted along the pipeline and received by acoustic sensors. This method succeeds in detecting and locating leaks when gas/liquid is introduced into the pipeline, thus not allowing for the continuous monitoring of the system during operation. Another important aspect to consider is its reliability, which is only achievable for short distances [16,17,18].

The method proposed in this paper is based on a non-invasive system composed of two pairs of acoustic sensors at the two extremities of the pipeline that acquire the signal to be processed for leakage surveillance. The surveillance implies two main directions: leak detection and localization. Detection is achieved using a pattern-based approach, while localization is rendered possible by advanced signal processing techniques that exploit signal deformations due to propagation in dispersive media such as the pipe wall. The proposed method allows for a continuous monitoring of thousands of meters of pipeline, as it is proved in the context of an in-service penstock.

This paper is structured as follows: Section 2 describes the theoretical background for leak detection and localization, Section 3 presents the results obtained in an operational scenario and, finally, Section 4 presents the conclusions of this paper and our future research work.

## 2. Materials and Methods

### 2.1. System Presentation

**Figure 1:**
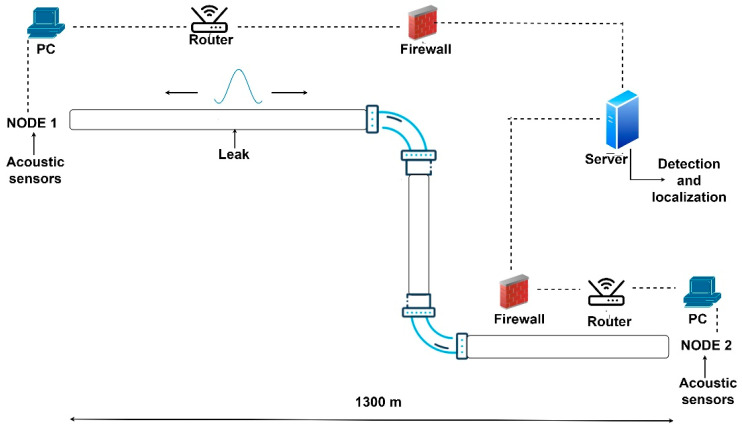
Schematic presentation of the system.

The system proposed in this paper is based on two sensing points, denoted as nodes in Figure 1, which are placed at the extremities of the pipe where access is usually possible and straightforward. Each node is composed of several non-intrusive sensors acoustic that cover the bottom section of the pipe. The assumption we make is that the leaks are generally located in the bottom part along the whole length of the pipe.

Each node contains a pre-amplifier stage to amplify the received signal and an acquisition part that will digitize the acoustic signals.

**Figure 2:**
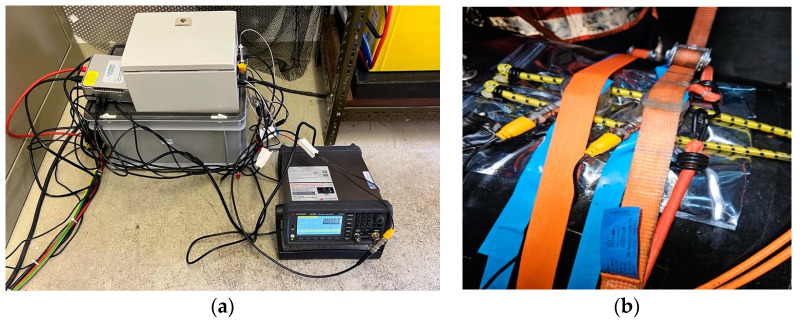
System at node 1: (**a**) the system used for data recording and data transmission to the server; (**b**) acoustic sensor placement.

**Figure 3:**
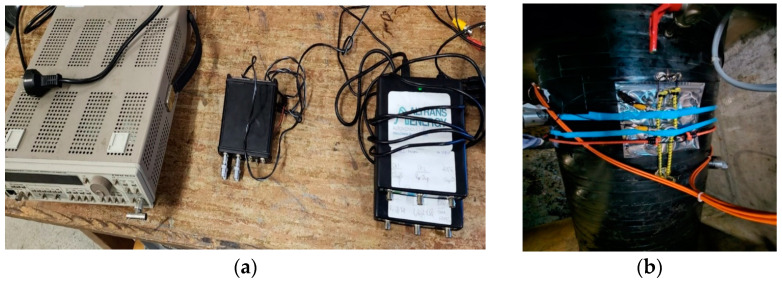
System at node 2: (**a**) the system used for data recording and data transmission to the server; (**b**) acoustic sensor placement.

Figure 2 and Figure 3 present pictures of the electronic devices serving as the sensing points at the extremities of the pipe and the acoustic sensors attached around the pipe section in both the upper and lower extremities.

In order to ensure the detection of the acoustic signals of interest, the acoustic sensors are composed of five piezoelectric sensors with a central frequency at 5 kHz. The sampling frequency is 20 kHz and allows for an increased time and frequency resolution, important for the localization of the leaks, as we will see later. The acquisition board is a HS-4 produced by TiePie Engineering, Sneek, The Netherlands.

Additionally, each node contains a computing unit in charge of pre-processing the acoustics signals, extracting the transients of interest and then transmitting the data to a server, where the signals from both nodes are processed. The next section presents the local processing algorithms at each node and also the server-side processing.

### 2.2. Leak Detection

The idea behind our algorithm is to exploit the acoustic transients that are generated when the water flow meets the positions where leaks occur. That is, when the water flow at high pressure meets a crack/hole responsible for a leak, a local transient is generated. This transient signal will propagate in both directions and will be acquired at the two nodes as shown in Figure 1.

**Figure 4:**
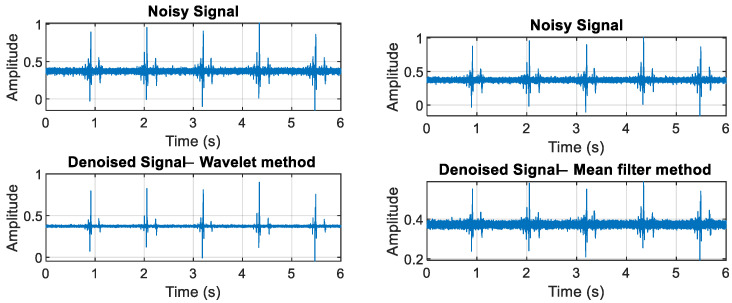
Signal denoising methods.

In our analysis, we implemented multiple denoising techniques, including wavelet denoising and the mean (average) filter method [19], to enhance signal quality and reduce noise interference, which is crucial for accurate data interpretation and analysis. As illustrated in Figure 4, wavelet denoising consistently outperformed other methods, providing superior results in terms of preserving signal integrity and minimizing distortion, thereby ensuring more reliable and precise outcomes [20,21,22,23].

**Figure 5:**
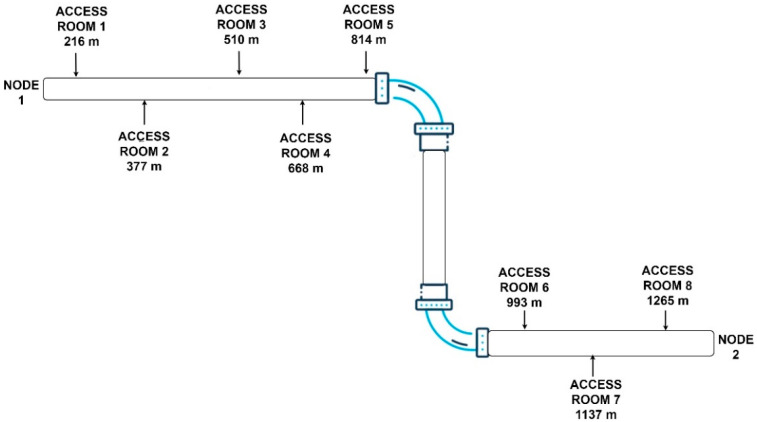
Schematic representation of the pipeline under analysis.

For leak detection, an individual database of corresponding signal patterns must be created for each pipe due to the individual characteristics of each pipeline. To accomplish this, leaks are introduced in a controlled manner using the purge valves placed in the access rooms, which are located at known distances along the pipeline shown in Figure 5. After collecting the patterns, the detection is performed with a matched filter that verifies if the pattern corresponding to a leak is present in the acquired signal.

In our detection approach, the propagation speed is assumed to be constant and its value represents the average propagation speed calculated by dividing the distance between the two sensing points by the time it takes for a wave emitted at one sensing point to propagate to the other sensing point located at the opposite end of the pipe. Knowing the pipe length and the propagation velocity, we compute the propagation time of the signal through the pipe using the formula below:

(1) $$t=\frac{L}{v_{prop}}$$

where t is the propagation time, L is the length of the pipe, which, in this case, is equal to 1300 m and $v_{prop}$ is the propagation speed approximated with 833 m/s.

**Figure 6:**
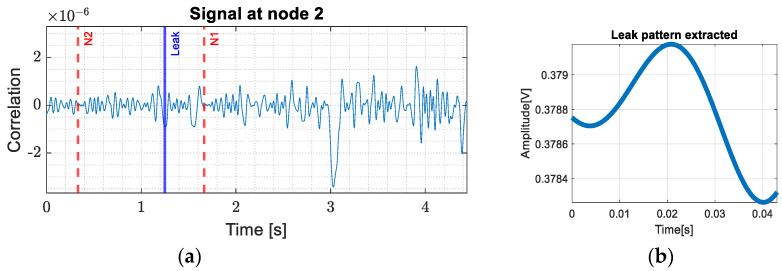
(**a**) Extraction of the leakage pattern from access room 3 at 3 L/min; (**b**) pattern extracted.

Using (1), it was determined that a timeframe containing the interference states of the whole pipe is represented in approximately 1.56 s. It is then possible to calculate the time moments corresponding to the leaks, knowing the position at which they occur. Leaks were introduced in access rooms two, three, four and five, each having two flow rates, 3 L/min and 6 L/min. The corresponding patterns for each controlled leak are determined from the acquired signals at node 2 as shown in Figure 6a. This figure depicts the signal acquired at node 2 for a 3 L/min leak in room 2, where we emphasize the timestamps corresponding to node 1 and 2 with red and the timestamp of the controlled leak with blue. Considering this approach, we constructed a database with 100 leak patterns for each room and each flow rate. This allowed us to have more perspectives on different types of leaks and their representative signals.

After creating the database to detect the presence of a pattern in the acquired signal, a matched filter was designed. The extracted pattern corresponding to a leak of 3 L/min for room two is shown in Figure 6b.

A matched filter y applied on a given signal s is a linear filter used where the impulse response is the time-reversed reference signal [24]:

(2) $$y[n]=\sum_{k=-\infty }^{\infty }x[k]\cdot h[n-k]$$

where h[n]=a⋅s[N−n], a is a constant and N is the sample corresponding to the reference time, in our case, the duration of the observation window. The s signals represent the leak patterns extracted during the controlled leakage tests and they are stored into our database in order to use them as reference signals. The matched filter application to an observed signal x is equivalent to the correlation of x with the reference waveform s. From this correlation, a decision threshold is applied to the matched filter output in order to determine if the reference signal s is present or not in the observed signal x.

### 2.3. Localization Principle

Once a transient is detected using matched filtering between the observed signals at each node and the references, the transient pattern is then transferred to the data server. Then, the analysis of the transients is required in order to localize their source. The challenge is that the moment in which the transient is generated is not known, as the leak can occur anywhere in the pipe. Also, the transients propagated and received at both nodes generally have different waveforms, which are more or less deformed according to the individual propagation paths.

For this reason, we focus the processing on using the advanced phase diagram representation [25,26]. The phase diagram representation has the advantage of being a data-driven method and not requiring any a priori model to analyze data, compared to the classic signal analysis methods such as wavelets [27,28]. This characteristic makes it suitable in our case as it offers a robust representation of transient signals, such as the ones coming from the leaks.

**Figure 7:**
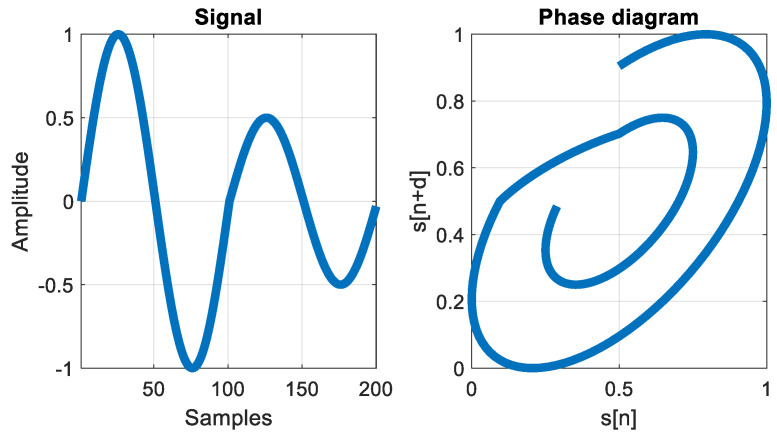
Signal and its phase diagram representation.

Starting from the time series corresponding to the signal of interest, (3), the way to construct the new representation space, the phase diagram, is highlighted in (4):

(3) x={x[1],x[2],…,x[N]}

(4) $$\vec{v_{\left[i\right]}}=\sum_{k=1}^{m}x[i+(k-1)d]*\vec{e_{k}},\;i=\bar{1,M}$$

where $\vec{v_{\left[i\right]}}$ are the phase space vectors, d is the delay between the samples, m represents the embedding dimension, N is the length of the time series, $\vec{e_{k}}$ are the axis unit vectors and M=N−(m−1)d [29]. An example of a signal and its phase diagram representation is presented in Figure 7.

The most important parameters are the delay, d, and the embedding dimension, m, giving the optimal representation of the phase diagram, where different methods are proposed for their choice [30].

**Figure 8:**
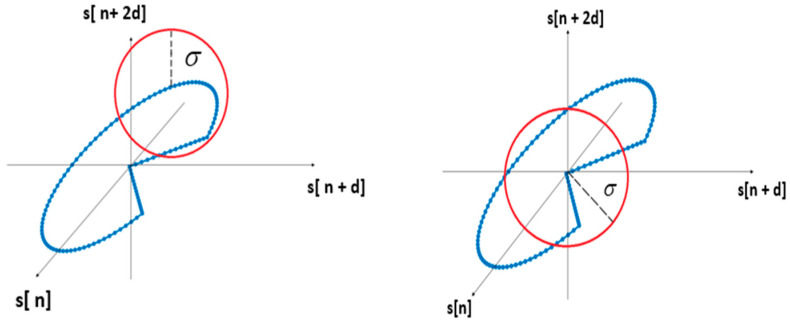
Representation of points close within a distance.

A feature of interest is the distribution of vectors and, more precisely, the distance between the phase diagram vectors because this gives us the shape of the trajectory [31]. For this, we want to determine the number of points which are close within a distance σ as shown in Figure 8. The parameter σ will give us the distance between vectors in the phase diagram. After that, we can calculate the ratio of near points to all vectors using (5):

(5) $$T_{j}(d,m,\sigma )=\frac{1}{N-(m-1)d}\sum_{j=1,j¹i}^{N-(m-1)d}\Theta (\left\|\vec{v_{\left[i\right]}}-\vec{v_{\left[j\right]}}\right\|-\sigma )$$

where Θ is Heaviside function, $\vec{v_{\left[i\right]}}$ is the distance vector of point i from diagram and $\left\|\vec{v}\right\|$ is the vector norm.

The number of points satisfying the condition to be within a distance σ is used below to determine the entropy.

(6) $$H(d,m,\sigma )=\frac{1}{N-(m-1)d}\sum_{j=1}^{N-(m-1)d}\log(T_{j}(d,m,\sigma ))$$

We perform the same process again for the next dimension m+1 to observe how much additional information we obtain from the new representation and define phase diagram-based entropy gradient (PDE) as in (7):

(7) PDE(d,m,σ)=H(d,m,σ)−H(d,m+1,σ)

**Figure 9:**
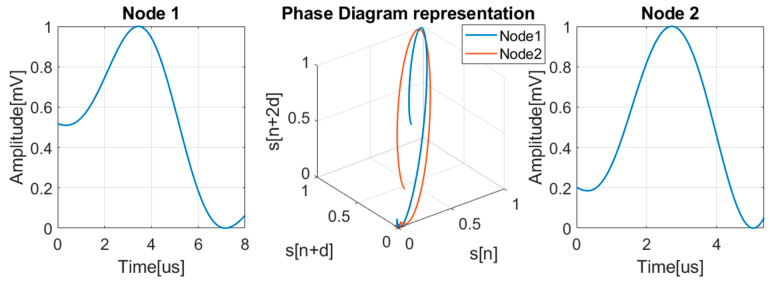
Phase diagram representation for the extracted patterns.

This algorithm is then applied to the transient received at the two ends of the pipe. We determine the phase diagram for the received signal at each node, which should emphasize similar features, although the original signals can be slightly different because of the propagation effects, as shown in Figure 9. Then, we compute the entropy and determine the relative distance of the leak as shown in (8)

(8) $$\frac{L_{1}}{L_{2}}=\frac{PDE_{1}}{PDE_{2}}$$

where $L_{i}$ represents the distance from node i to leak and $PDE_{i}$ is the phase diagram-based entropy of the signal acquired at node i, where $i\in \left\{1,2\right\}$.

The fact that this metric is data-driven is the most important advantage brought forth by our method. As it is not dependent on any model, the phase diagram-based entropy may be used in any type of signal analysis.

One of the conceptual limitations of the data-driven method is its reliance on identifying useful parts of signals or data based on their coherence in representation domains, such as the phase diagram in our case. The abstract notion of coherence depends heavily on the nature of the data-driven representation. In our context, coherence is defined by the smooth variation of trajectories, which can be too general for finely discriminating different transient parts of the signals. Therefore, post-processing operations are necessary to achieve better discrimination between transients.

In our application context, the data-driven technique requires the creation of a database by generating controlled leaks at various positions along the pipe. If this is not feasible, databases from other pipes can be used, but the results may not be optimal.

## 3. Experimental Results

The experiment was conducted on an in-service penstock of L=1300 m. The schematic of the pipeline is presented in Figure 4. The objective of this experiment was to demonstrate the capacity of our method to detect and localize leaks.

**Figure 10:**
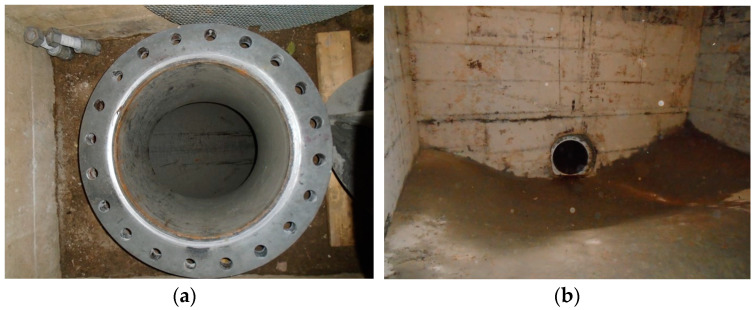
(**a**) Access room 2 used for controlling leak; (**b**) starting point of water pipeline.

Firstly, our system continuously monitored the penstock while controlled leaks were introduced. Controlled leaks refer to manually opening a valve such that we know the exact position and flow rate of the leak along the pipe. Figure 10a presents the room in which the operators performed the controlled leak. This allowed us to acquire and determine the patterns which we later used to detect other leaks that might appear in time.

The continuous monitoring of the in-service penstock led to the detection of several other uncontrolled leaks, which were further validated in an offline manner using a robot and a video camera that was introduced inside the emptied pipe. Figure 10b shows the start of the pipeline when it was out of service in order to allow for the robot-based inspection.

### 3.1. Experimental Results

**Figure 11:**
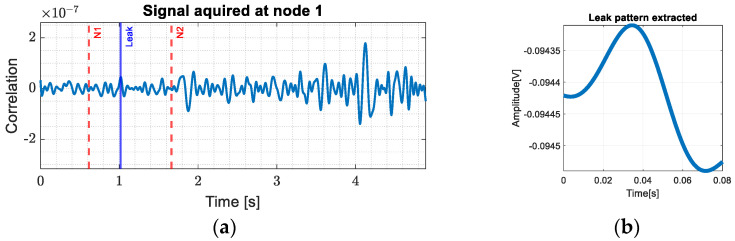
(**a**) Leak detection from signal acquired at node 1; (**b**) the pattern determined.

**Figure 12:**
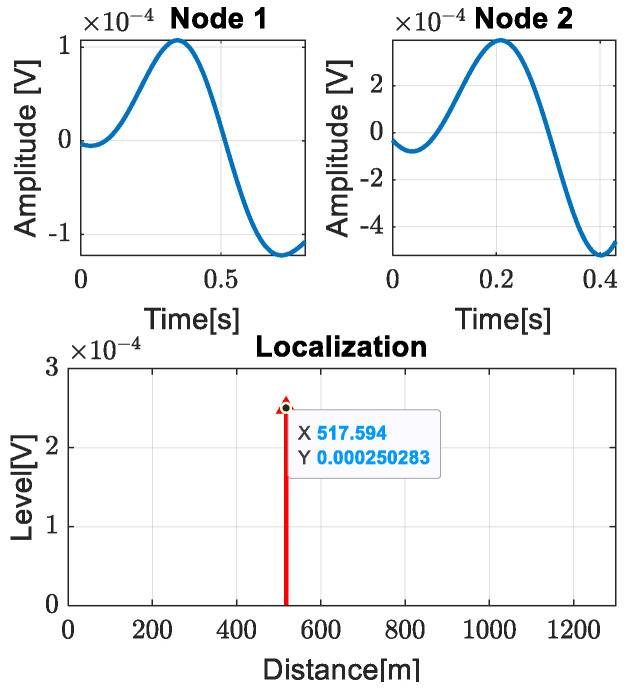
Localization of the leak.

In this section, we present the entire process of acquiring data for both nodes and the subsequent processing steps that allow for leak detection and localization. The signal acquired at node 1 was filtered with a lowpass filter and the result is presented in Figure 11a. After that, we applied the matched filter to detect the leak. The pattern identification and the leak localization are presented in Figure 11b and Figure 12.

Leak localization was performed based on the entropy, as in (8), while the *Level* of the component was estimated as the average value of the maximum amplitude of the patterns identified for node 1 and node 2. As shown in Figure 12, it can be seen that the leak is 517 m away from node 1. From the pipe plan, shown in Figure 5, we can see that access room three is at a distance of 510 m from node 1, resulting in a leak localization accuracy of 7 m.

**Figure 13:**
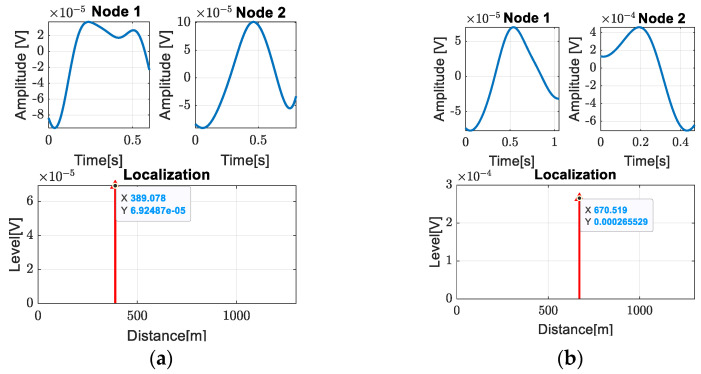
Leak detection for (**a**) access room 2; (**b**) access room 4.

This process was repeated for all the other rooms. Figure 13 shows the localization results for the data acquired when the leak was at access room 2 and 4. These access rooms are at distances of 377 m and 668 m from node 1. As can be seen, our proposed method provides very good accuracy with respect to the experimental analyzed data.

### 3.2. Continuous Monitoring Results

In this section, we present the results of our method for the continuous monitoring of the penstock of the Leytegeon hydroplant exploited by OIKEN. The acoustic monitoring system has been in use since December 2023 and several leak points have been identified and surveyed. In order to confirm these monitoring results, a video inspection was conducted on 10 February 2024. The next figures illustrate the equipment used for this inspection.

Based on the experimentally created database, a daily analysis of the pipeline under normal operating conditions was carried out, performing a synchronized acquisition at the two nodes and then processing the data using the proposed method.

**Figure 14:**
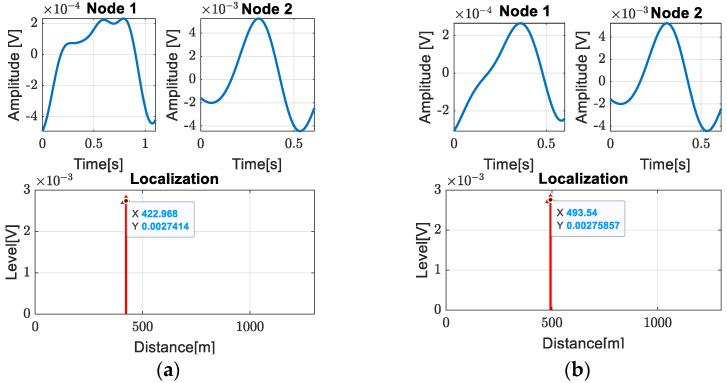
Localization of (**a**) first leak; (**b**) second leak.

**Figure 15:**
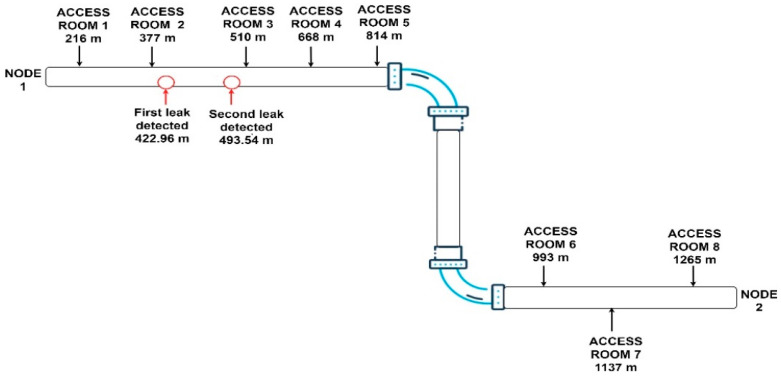
Schematic representation of the pipeline with detected leak positions.

With our method, beside the artificial leakages, two new leaks were detected and located at distances of 422 m and 493 m from node 1, as shown in Figure 14 and Figure 15.

**Figure 16:**
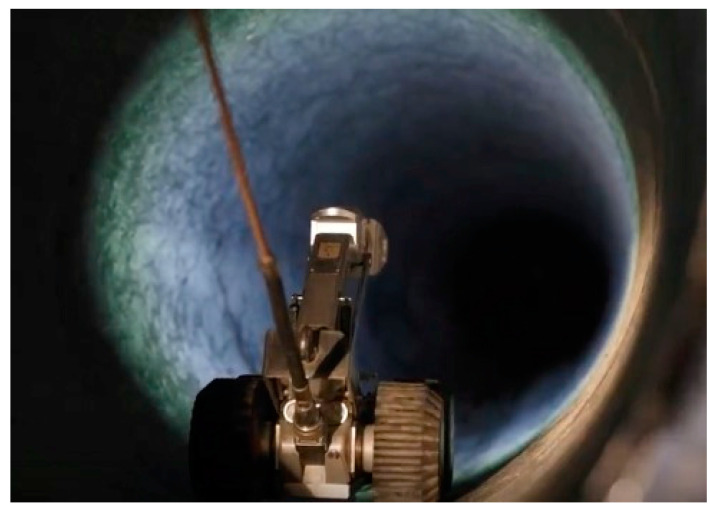
The robot used for video surveillance.

**Figure 17:**
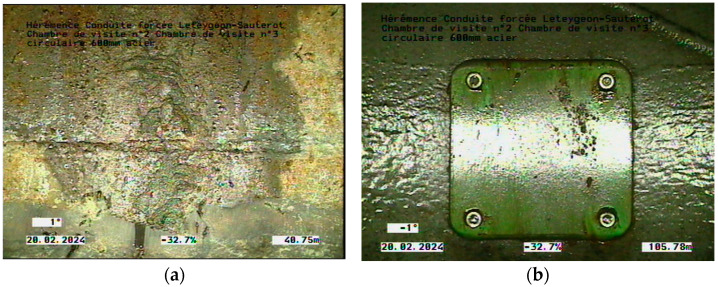
Video surveillance results: (**a**) first localised leak; (**b**) second localised leak.

To validate our method, a robot with a surveillance camera attached to it was introduced, as presented in Figure 16, taking separate video clips for consecutive rooms. The distance displayed on the video represents the distance between the two rooms. Figure 17 present the images of the leaks from the pipeline extracted after analyzing the video.

The images extracted from video surveillance are between access room 2, which is 377 m away, and access room 3, which is 510 m away from node 1. Therefore, we can easily calculate the distance of the first leak from node 1 as $D_{1}\;=\;377\;m\;+\;40.75\;m\;=\;417.75\;m$ and the second leak at $D_{2}\;=\;377\;m\;+\;105.78\;m\;=\;482.78\;m$. As can also be seen in this case, the leaks were detected with very high accuracy, which confirms the efficiency of the proposed method.

An overview of the results is presented in Table 1 and Table 2.

As can be observed from the previous tables, the locations of the leaks were determined with high accuracy, regardless of the distance from the leak. This highlights the fact that the method is not dependent on pipe length.

## 4. Conclusions

Leaks have a great impact, both economically and environmentally, making their detection and localization a critical problem. The fact that the pipeline network is largely underground makes the localization much more difficult to achieve and reduces the efficiency of some methods. Current methods have a number of important disadvantages: they are intrusive, accurate just for short pipelines and highly expensive.

This paper presents a non-intrusive method for leak detection and localization in pipelines. The advantages of this method are as follows: it is low-cost, acoustic sensors can be easily placed at the ends of the pipe, and the method works regardless of the length of the pipeline and provides good accuracy.

Our future work is based on quantifying leak levels and using artificial intelligence to classify them.

## Figures and Tables

**Table 1:** Leaks localization and relative error.

Leak	Our Proposed Method	Position of Access Rooms	Relative Error
Leak at access room 2	398.078 m	377 m	0.928%
Leak at access room 3	517.594 m	510 m	0.583%
Leak at access room 4	670.519 m	668 m	0.193%

**Table 2:** Localization of leaks performed in a controlled manner in the access rooms.

Leak	Our Proposed Method	Video Surveillance Results	Relative Error
Leak 1	422.96 m	417.75 m	0.4%
Leak 2	493.54 m	482.78 m	0.82%

## Data Availability

The data presented in this study are available upon request from the corresponding author.
